# Relationships among Early Adversity, Positive Human and Animal Interactions, and Mental Health in Young Adults

**DOI:** 10.3390/bs11120178

**Published:** 2021-12-14

**Authors:** Kerri E. Rodriguez, Shelby E. McDonald, Samantha M. Brown

**Affiliations:** 1 Human-Animal Bond in Colorado (HABIC), School of Social Work, College of Health and Human Sciences, Colorado State University, Fort Collins, CO 80523, USA; 2 Clark-Hill Institute for Positive Youth Development, Virginia Commonwealth University, Richmond, VA 23284, USA; smcdonald3@vcu.edu; 3 School of Social Work, College of Health and Human Sciences, Colorado State University, Fort Collins, CO 80523, USA; samantha.brown@colostate.edu

**Keywords:** childhood adversity, adverse childhood experiences (ACEs), mental health, companion animals, human–animal interaction

## Abstract

Adverse childhood experiences (ACEs) are associated with poor mental health. Emerging research demonstrates the protective role of positive childhood experiences, including a positive sense of self and relationships with both humans and animals, in mitigating the impacts of early life adversity on mental health outcomes. This study examined whether benevolent childhood experiences (BCEs) or relationships and interactions with pets during childhood moderated the link between ACEs and current mental health symptoms in a sample of young adults. Students (*N* = 214) recruited from a public university in the U.S. completed an online survey. The results showed that ACEs were significantly associated with worse mental health symptoms, including anxiety and depression. Neither emotional closeness to a childhood pet dog nor positive interactions with a childhood pet were significant moderators of the relationship between ACEs and mental health. In contrast, more BCEs were associated with better mental health, and their interaction with ACEs was significant such that adversity-exposed young adults with high BCEs reported fewer mental health symptoms than those with low BCEs. The results highlight the need for continued research on differential experiences that may be protective in the relationship between adversity exposures and mental health.

## 1. Introduction

Adverse childhood experiences (ACEs) are stressful events that occur before age 18 such as abuse, neglect, and household challenges [1]. It is estimated that 61% of adults have experienced at least one ACE, while 25% experience three or more ACEs [2]. Experiencing these adversities during childhood can lead to lasting negative effects on health and wellbeing [3], and when multiple ACEs co-occur, their cumulative impact exacerbates poor outcomes [4]. Recent research suggests that in addition to conventional ACEs, such as exposure to abuse, neglect, and household challenges, other early adversities that extend beyond the household such as discrimination, bullying, or neighborhood violence may also impact long-term health outcomes [5]. The accumulation of ACEs is significantly related to most of the leading causes of death, as well as a wide breadth of psychosocial outcomes related to poor health and wellbeing in adolescence and adulthood [6].

Given the deleterious impacts of ACEs on health and wellbeing, research has recently begun to identify protective factors that can help individuals overcome the negative impacts of ACEs on mental health outcomes [7,8]. Indeed, a positive sense of self and relational experiences with caregivers, peers, and teachers—known as benevolent childhood experiences (BCEs)—can mitigate the consequences of ACEs on health and wellbeing [9,10,11]. For example, a 2020 study among adolescents found that when both ACEs and BCEs were considered together to explain health and wellbeing, cumulative ACEs were not significantly associated with any health indicators (indicating a protective effect of BCEs; [12]). In addition, social support in the form of nurturing, positive relationships with parents, family members, and peers can promote adaptive outcomes among individuals experiencing adversity [13] and has been found to moderate the relationship between ACEs and later health outcomes [14].

In addition to supportive human relationships, there is emerging evidence to suggest that non-human social support in the form of a relationship with a pet, or companion animal, may also serve as a protective factor in the context of ACEs. Companion animals can satisfy children’s need for comfort and reassurance [15,16], and dogs, in particular, can serve as attachment figures during childhood [17,18]. In fact, research suggests that children of single-parent homes [19] and children with no siblings [20] report greater attachment to companion animals than children in two-parent households and children with siblings, suggesting that children may turn to animal companionship for social support. Despite limited causational research quantifying the potential protective role that positive child–animal relationships play in the face of adversity, few cross-sectional studies have found that children form strong attachment relationships with companion animals in the context of certain adversities such as living in foster care [21], experiencing parental divorce [22], and witnessing familial conflict [23]. In addition, a 2012 study found that college-aged females who had been neglected during childhood reported more attachment to their current pets than females who had not been neglected, suggesting that pets may serve as important sources of social support for adversity-exposed individuals throughout the lifespan [24].

Positive relationships with childhood pets may also moderate the relationship between ACEs and later health outcomes, although there has been little research in this area. A 2019 study found that positive engagement with a pet in the home significantly moderated the relationship between exposure to intimate partner violence (e.g., physical or psychological harm among spouses/intimate partners) and internalizing anxious/depressive and posttraumatic stress symptoms among children aged 7 to 12 years [25]. In addition, a 2021 study of sexual and gender minority emerging adults (aged 18–21 years) found that high levels of emotional comfort derived from pets buffered the negative impact of lifetime gender-based victimization on self-esteem, regardless of whether one perceived high or low levels of social support from humans [26]. However, there is a lack of research examining the potential protective role that child–pet relationships play in explaining mental health symptoms in young adulthood. It is also unknown how positive relationships with childhood pets may complement other BCEs to potentially moderate the relationship between ACEs and later health outcomes. In this study, we chose to examine pet relationships during childhood broadly as well as child–dog relationships specifically. This is due to the fact that children report higher attachment to pet dogs compared to other pets [20] and because of findings from observational studies suggesting physiological stress-buffering effects from both therapy dogs [17,27,28] and pet dogs [27,28] for children and adolescents.

The objective of the current study was to evaluate the potential protective role of positive experiences with both humans and animals in mitigating the relationship between childhood adversity and poor mental health. Specifically, we examined both the main and interactive effects of ACEs and positive childhood experiences, including positive sense of self and relationships with both humans and animals, on self-reported mental health symptoms of young adults. This key developmental period was chosen due to this time being an important transitional period often characterized by significant stressors and mental health challenges associated with ACEs [29]. We had two hypothesis-driven aims in this study. First, we hypothesized that cumulative ACE exposures would be significantly related to self-reported mental health symptoms among young adults, replicating previous studies (e.g., [30,31]). Second, we evaluated the potential protective role of positive childhood experiences, conceptualized via BCEs and relationships and interactions with pets during childhood, in moderating the relationship between ACEs and mental health symptoms. We hypothesized that the relationship between ACEs and young adults’ self-reported mental health symptoms will be significantly moderated by the frequency of child–pet interactions, the strength of the child–dog bond, and BCEs, independently.

## 2. Materials and Methods

### 2.1. Participants and Procedures

All protocols were approved by the Colorado State University Institutional Review Board (Protocol #2142). Participants were undergraduate and graduate students over the age of 18 years recruited in March–August 2021 from a large public university in the United States. Recruitment occurred through the Psychology Department and Human Development and Family Studies Department research pool such that student participants received partial course credit for their participation. Informed consent was obtained electronically by asking student participants to agree to participate before beginning the online survey. Participation was voluntary, and participants were told they could choose to not answer any questions in the online survey. To ensure that participants were attending to items, two attention checks were used. The first asked participants to type in the word “yellow” in a text box roughly halfway through the survey. The second was an exaggerated statement not relating to study items (“Eating glass for breakfast”) inserted into a matrix-style question block towards the end of the survey.

Sample demographics are displayed in Table 1. The final sample included *N* = 214 young adult participants who completed surveys. Of the 239 individuals who began the survey, *n* = 9 were excluded because they were not over 18 years of age, *n = 9* were excluded from not passing attention checks, and *n* = 7 were excluded due to survey incompleteness. The sample was mostly single (92%), female (79%), White (80%), and not of Hispanic origin (80%), with a mean age of 19.88 years (ranging from 18–37 years).

### 2.2. Measures

Demographic variables were collected via self-report questions asking about age, gender identity, relationship status, education level, race/ethnicity, and pet ownership. To assess pet ownership, participants were asked if they had pets growing up with check all that apply choices of “yes—pet dog(s)”, “yes—pet cat(s)”, “yes—other pet(s)” in which they could indicate the specific type of pet, or “no”.

ACEs were assessed using a broadened ACEs scale building on the work from Felitti et al. [1] and Cronholm et al. [5]. Participants were asked to retrospectively self-report if they experienced a list of 15 conventional and expanded ACEs before the age of 18 years (“yes” or “no”): (1) physical abuse, (2) emotional abuse, (3) sexual abuse, (4) physical neglect, (5) emotional neglect, (6) parental divorce/separation, (7) witnessed intimate partner violence, (8) incarcerated household member, (9) substance-using household member, (10) mentally ill household member, (11) lived in an unsafe or high-crime neighborhood, (12) witnessed violence, (13) experienced racism/discrimination, (14) experienced bullying, and (15) lived in foster care. A cumulative ACEs score was calculated based on summing “yes” answers such that a higher score indicated more ACEs, with scores ranging from 0 to 15. Internal reliability for this ACEs measure was acceptable (McDonald’s omega = 0.80).

BCEs were assessed using the Benevolent Childhood Experiences scale (BCEs; [9]). The BCEs scale is a 10-item checklist asking participants to answer “yes” or “no” to 10 different positive experiences that may have been present before age 18 years: (1) had at least one caregiver with whom they felt safe, (2) had at least one good friend, (3) had beliefs that gave them comfort, (4) liked school, (5) had at least one teacher who cared about them, (6) had good neighbors, (7) had an adult who could provide them with support or advice, (8) had opportunities to have a good time, (9) liked themselves or felt comfortable with themselves, and (10) had a predictable home routine. A BCEs score was calculated by summing “yes” answers, such that a higher score represented more benevolent childhood experiences, with scores ranging from 0 to 10. Internal reliability for this BCEs measure was acceptable (McDonald’s omega = 0.74).

Mental health symptoms were assessed via the Brief Symptom Inventory (BSI; [32]). The BSI is a 53-item self-report survey assessing the current severity of a range of mental health symptoms on a Likert scale from 0 (not at all) to 4 (extremely). The BSI contains nine subscales including somatization, obsessive compulsive, interpersonal sensitivity, depression, anxiety, hostility, phobic anxiety, paranoid ideation, and psychoticism. Items were summed and averaged to create a Global Severity Index (GSI) score, reflecting both the number of symptoms present and the severity of each symptom experienced. For the purposes of this research, we also calculated raw scores for the subscales of Anxiety and Depression. Raw GSI scores as well as Anxiety and Depression subscale scores were converted to T scores according to a gender-keyed adult nonpatient norm group [33]. T-scores were normalized such that the population mean is 50 with a standard deviation of 10, with higher scores indicative of worse mental health symptoms. Internal reliability for the BSI was high (McDonald’s omega = 0.98).

Participants that reported having any pet (“cat(s)”, “dog(s)”, or “other”) during childhood were given a 12-item version of the Children’s Treatment Towards Animals (CTAQ) scale [34], a self-report questionnaire designed to assess children’s humane treatment of animals. Participants were asked to retrospectively indicate the frequency in which they engaged in different positive behaviors with their pet(s) (e.g., “talk to”, “play with”, “pet”). Responses were scored such that higher scores reflected higher levels of positive interactions with childhood pets, with scores ranging from 12 to 36. Internal reliability for the modified CTAQ was good (McDonald’s omega = 0.83).

Participants that reported having a pet dog(s) during childhood were given the Monash Dog–Owner Relationship Scale (MDORS; [35]) 10-item Emotional Closeness subscale to measure the emotional closeness between the individual and their childhood pet dog. If the participant had more than one pet dog during childhood, participants were asked to report on their relationship with the pet dog to which they felt “closest to”. Participants were asked to retrospectively report on their agreement regarding the strength of their relationship with their childhood pet dog (e.g., “If everyone else left me, my dog would have still been there for me”; “I wished my dog and I never had to be apart”). After reverse scoring one item, scores of individual items were summed such that higher scores reflected stronger emotional closeness, with scores ranging from 10 to 50. Internal reliability for the MDORS EC subscale was excellent (McDonald’s omega = 0.93).

### 2.3. Statistical Analyses

All analyses were conducted in SPSS Version 27.0 (IBM Corp. Armonk, NY, USA). First, descriptive analyses were conducted to describe demographic and sample characteristics, and zero-order correlations were conducted between key study variables. Due to small cell sizes for specific gender identity groups, gender identity was recoded into female, non-binary, or self-identified/other = 1 and male = 0. To address our primary hypotheses, three separate multiple regression analyses were run with mental health symptoms T-Scores (BSI-GSI) as the dependent variable, controlling for age (continuous), gender identity (dichotomous), and ethnicity (dichotomous, Hispanic/Latinx = 1, Non-Hispanic/Latinx or prefer not to answer = 0). The first regression model included cumulative ACEs score and child–dog emotional closeness (MDORS) as main effects variables and an ACEsxMDORS interaction variable. The second model included cumulative ACEs score and positive child–pet interactions (CTAQ) as main effects variables and an ACEsxCTAQ interaction variable. The third model included cumulative ACEs and BCEs scores as main effects variables and an ACEsxBCE interaction variable. An additional sensitivity analysis was conducted on the first and second models in which BCEs were added as an additional covariate. To further explore the nature of significant interactions, we conducted simple slopes analyses, and scores were plotted at one standard deviation above and below the mean. Sensitivity analyses also included a replication of these three models with separate dependent variables of the BSI Anxiety and Depression subscale T-Scores. A post-hoc power analysis conducted using G*Power [36] determined that the achieved sample size (*N* = 214) was sufficient to achieve power of 0.99 to detect a medium effect (*f*^2^ = 0.15) at an error probability of α = 0.05 with 6 independent variables (critical *F* value = 2.05).

## 3. Results

The descriptive statistics and zero-order correlations between key study variables are displayed in Table 2. The participants endorsed experiencing an average of 2.20 of 15 ACEs (*SD* = 2.59; ranging from 0–11), the most common of which were having a mentally ill household member (28%) and being bullied (27%). Participants endorsed experiencing an average of 8.97 of 10 BCEs (*SD* = 1.57; ranging from 2–10). Participants reported higher than average mental health symptoms in comparison to an adult, nonpatient sample (*M* = 59.56, population *M* = 50.00 [33]). Young adults’ current mental health symptoms, including anxiety and depression symptoms, were positively related to ACEs and negatively related to BCEs with moderate strength. ACEs were not significantly correlated with child–dog emotional closeness nor child–pet positive interactions. ACEs were negatively related to BCEs. Finally, there was a strong positive correlation between child–dog emotional closeness and child–pet positive interactions.

**Figure 1:**
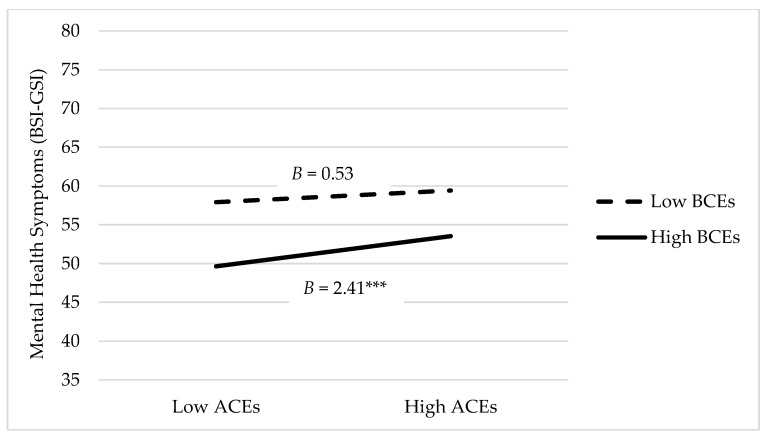
Moderating effect of BCEs on the association between ACEs and mental health symptoms. BSI-GSI, Brief Symptom Inventory Global Severity Index; BCEs, Benevolent Childhood Experiences; ACEs, Adverse Childhood Experiences; *B =* Unstandardized beta; *** *p* < 0.001.

Table 3 displays the outputs from the main effects and moderation analyses. Controlling for age, gender identity, and ethnicity, ACEs were significantly related to mental health symptoms such that a higher cumulative ACEs score was associated with a higher BSI GSI score and thus more mental health symptoms (*p* < 0.001). There were no significant interaction effects between ACEs and child–dog emotional closeness (Model 1) or ACEs and child–pet positive interactions (Model 2) on mental health symptoms. A sensitivity analysis was performed by adding BCEs as an additional covariate to these models, which did not alter the significance level of any main or interactive effects. In Model 3, there was a significant interaction effect between ACEs and BCEs on mental health symptoms such that the relationship between ACEs and mental health symptoms was dependent on the level of BCEs reported. Figure 1 displays the mean levels of mental health symptoms for those with low (*M*—1*SD*) and high (*M* + 1*SD*) levels of ACEs at low (*M*—1*SD*) and high (*M* + 1*SD*) levels of BCEs. Figure 1 illustrates that those with high BCEs and low ACEs report better mental health in young adulthood than those with low BCEs and high ACEs.

Additional sensitivity analyses were performed replicating these models with BSI anxiety and BSI depression T-Scores as the dependent variables. The results confirmed that there were no significant interaction effects between ACEs and child–dog emotional closeness (*B* = −0.02, *p* = 0.790) nor ACEs and child–pet positive interactions (*B* = 0.01, *p* = 0.950) on anxiety symptoms. Similarly, there were no significant interaction effects between ACEs and child–dog emotional closeness (*B* = −0.04, *p* = 0.470) nor ACEs and child–pet positive interactions (*B* = 0.02, *p* = 0.746) on depression symptoms. However, there were significant interaction effects between ACEs and BCEs on both anxiety symptoms (*B* = 0.36, *p* = 0.031) and depression symptoms (*B* = 0.55, *p* < 0.001) such that those with high BCEs and low ACEs reported fewer anxiety and depression symptoms in young adulthood than those with low BCEs and high ACEs.

## 4. Discussion

The purpose of this study was to examine both the main and interactive effects of ACEs and positive childhood experiences, including positive sense of self and relationships with both humans and animals, on self-reported mental health outcomes of young adults. Overall, the results indicate a direct relationship between ACEs and current mental health symptoms of young adults that is significantly moderated by BCEs but not positive child–pet or child–dog relationships. These relationships held for a global measure of mental health symptoms as well as specific anxiety and depression symptoms. These preliminary findings have implications for future research on understanding the relevance of protective factors in the relationship between childhood adversity and subsequent health and wellbeing outcomes.

The recruited sample of mostly White, non-Hispanic women reported relatively low levels of childhood adversity. Nonetheless, results indicated that cumulative ACEs had a significant effect on self-reported mental health, even after controlling for covariates. This confirmed our first hypothesis and replicates other recent studies which have found significant relationships between cumulative ACEs and depression and anxiety [37], health-related quality of life, [38] and overall stress [29] among college-aged young adults.

The second aim of this research was to evaluate the potential protective role of positive childhood experiences, including BCEs and positive child–pet and child–dog relationships, in moderating the association between cumulative ACEs and mental health symptoms. Although those with more ACEs tended to report worse mental health symptoms, the results indicate that BCEs significantly moderated the relationship between ACEs and mental health. Specifically, those that experienced more BCEs in parallel with adversity had better mental health outcomes (including overall mental health as well as anxiety and depression) than those who experienced fewer BCEs. This is consistent with recent findings that BCEs can significantly reduce the impact of ACEs on subsequent mental health [10,12,39] and suggests that future research should consider positive childhood experiences when elucidating the impact of childhood adversity on mental health and wellbeing in young adulthood.

We also evaluated the potential protective role of positive child–pet relationships and the child–dog bond in moderating the relationship between cumulative ACEs and mental health. There were no significant correlations between child–pet relationship variables and ACEs, such that participants who reported more ACEs did not report being more or less emotionally close with their pet dogs, nor did they report having more or less positive interactions with their pets in general. This is in contrast with research suggesting that children may develop strong relationships with pets in times of adversity (e.g., [21,23]). There were also no significant correlations between child–pet relationship variables and BCEs, which suggests that positive child–pet interactions may be independent of positive interactions with peers, adults, and teachers. Finally, refuting our hypotheses, child–dog emotional closeness and child–pet positive interactions did not significantly moderate the relationship between cumulative ACEs and mental health (including overall mental health as well as anxiety and depression). This suggests that these positive experiences, in contrast to BCEs, did not serve as significant protective factors in this population. In other words, adversity still has a strong relationship with mental health in young adulthood regardless of the retrospective strength of the child–dog relationship or child–pet interactions.

One possible explanation for why we may not have found significant moderating effects of child–pet relationships in the context of adversity concerns the measurement of childhood adversity in this study. Prior studies that have found protective effects of child–pet relationships on associations between childhood adversity and mental health have used continuous measures of specific forms of victimization (e.g., exposure to intimate partner violence), whereas this research used a cumulative ACEs approach. The cumulative ACEs approach has garnered widespread use and treats each form of victimization as equal, assuming an equal ‘dose–response’ relationship between the total score and mental health outcomes [40]. Although there is robust support for the use of this theoretical conceptualization of adversity, particularly in relation to explaining mental health outcomes, prior studies also indicate that it is important to distinguish between specific forms (e.g., physical abuse vs. incarcerated parent) and dimensions (threat vs. deprivation) of adversity when evaluating factors that can attenuate the deleterious impacts of adversity [39,41]. Therefore, more research is needed to understand the role of child–pet relationships with respect to both cumulative and specific forms of adversity.

Another second consideration in explaining these null findings is that we did not measure the potential negative aspects of the child–pet relationship, such as the presence of animal abuse in the home. Research has found that in homes where interpersonal violence (e.g., child abuse, intimate partner violence) and substance use exists, animal abuse is also more likely to exist [42,43]. For example, in contexts in which a child–dog bond was strong, but the dog may have been maltreated, the child–dog relationship may not have been contributing to a positive childhood experience. However, recent research suggests that social support from a pet can buffer the effects of family violence on health outcomes for children, even when animal cruelty is occurring [44]. Consequently, failing to measure and subsequently control for animal cruelty exposure may have limited our findings by resulting in unaccounted variability. Future research should incorporate the assessment of both negative and positive aspects of the child–pet relationship in moderation analyses.

Finally, it is possible that we were unable to detect a protective effect of child–pet relationships on mental health due to the types of ACEs that were most prevalent in our sample. The most prevalent ACEs in our sample were having a mentally ill (28%) or substance-using household member (22%), experiencing emotional neglect (22%), and experiencing bullying (27%). These forms of childhood adversity typically involve less perceived threats when compared to direct forms of victimization, such as exposure to intimate partner violence or physical abuse. Indeed, positive human–animal interactions are hypothesized to promote wellbeing by buffering stress both prior to and after activation of the stress response system [45,46] and by providing socioemotional support that aids pet owners in perceiving potential stressors as less threatening [47]. For example, a recent 2021 study found that emotional comfort derived from pets is associated with mental wellbeing (i.e., self-esteem) at moderate and high levels of victimization but not low levels [48]. Research on diverse samples with a broad range of ACEs is needed to better understand how child–pet relationships and ACEs interact to influence mental health in childhood throughout young adulthood. Therefore, we recommend that future studies assess the benefits of pet–child relationships in relation to cumulative ACEs and with respect to specific forms and dimensions of childhood adversity.

### Limitations

This study is not without its limitations. First, the recruited population in this study was a convenience sample recruited from a single public university, in which most participants were White, non-Hispanic, and female-identifying. The homogeneous sample limits the generalizability of the research findings. In addition, due to sample homogeneity, we were unable to explore associations among different sociodemographic groups, such as by race, income, or relationship status. Second, outcomes were collected via self-report measures that asked participants to retrospectively report on their positive and negative childhood experiences. This method of data collection may be limited by recall biases, in which participants may have skewed or inaccurate memories of their childhood experiences, as well as social desirability biases, in which participants may be reluctant to accurately report their traumatic experiences [49]. We also did not quantify emotional closeness relationships among children and non-dog pets, such as cats, small mammals, and/or reptiles or fish. In addition, individuals were only asked about their relationship with their own pets, which may have overlooked positive engagement and emotional closeness with animals that were not the participant’s pet but were still an important part of the child’s social ecology.

## 5. Conclusions and Implications

In conclusion, this study found that young adults who experienced ACEs are at greater risk for poor mental health, but that BCEs can play a protective role in buffering the relationship between ACEs and mental health symptoms in young adulthood. Our results do not support prior research suggesting that children may develop strong relationships with pets in times of stress or adversity or that child–pet interactions have a protective role in the association between childhood adversity and mental health. However, our findings are limited by a homogenous sample, retrospective reporting, and a cumulative conceptualization of adversity. Future research employing longitudinal methods will be particularly valuable given that social support from peers, parents, and siblings may continue throughout young adulthood, whereas the social support provided by a pet is more likely to be short-lived and potentially complicated by loss over time. In addition, studies are needed to identify whether there are sensitive periods during which child–pet relationships and BCEs are most impactful in attenuating the relationship between ACEs and poor mental health in childhood and young adulthood. Together, this research suggests that there is an important need to examine broad childhood adversities in addition to protective factors, namely perceived support from family, friends, and pets, which may mitigate the effects of adversity on mental health symptoms. Such an understanding may elucidate important targets for intervention to promote positive health outcomes across the lifespan.

## Figures and Tables

**Table 1:** Demographics of *N* = 214 participants.

	*M (SD)* or *N* (%)
Age (in years)	19.88 (2.59)
Relationship Status	
Single, never married	197 (92.06%)
Married or domestic partnership	15 (7.00%)
Divorced	1 (0.47%)
Prefer not to answer	1 (0.47%)
Gender Identity	
Female	170 (79.44%)
Male	37 (17.29%)
Other or self-identified	4 (1.87%)
Non-binary	3 (1.40%)
Prefer not to answer	0 (0.00%)
Ethnicity	
Not of Hispanic origin	172 (80.37%)
Hispanic origin	35 (16.36%)
Prefer not to answer	7 (3.27%)
Race	
White	171 (79.91%)
Multiple races	17 (7.95%)
Asian	10 (4.67%)
Black or African American	6 (2.80%)
Native Hawaiian or Pacific Islander	2 (0.93%)
American Indian or Alaska Native	1 (0.47%)
Prefer not to answer	7 (3.27%)
Education Status	
First-year student	121 (56.54%)
Second-year student	49 (22.90%)
Third-year student	28 (13.08%)
Fourth-year student	13 (6.08%)
Fifth-year or more student	2 (0.93%)
Graduate student	1 (0.47%)
Pet Ownership During Childhood ^a^	
Yes, Dog(s)	177 (82.71%)
Yes, Cat(s)	83 (38.79%)
Yes, Other	75 (35.05%)
No	10 (4.67%)
ACEs ^a^	
Mentally ill household member	59 (27.57%)
Experienced bullying	58 (27.10%)
Parental divorce/separation	51 (23.83%)
Emotional abuse	48 (22.43%)
Emotional neglect	46 (21.50%)
Substance using household member	47 (21.96%)
Felt discrimination	38 (17.76%)
Incarcerated household member	24 (11.21%)
Physical abuse	23 (10.75%)
Sexual abuse	20 (9.35%)
Crime neighborhood	20 (9.35%)
Unsafe neighborhood	14 (6.54%)
Witnessed domestic violence	10 (4.67%)
Physical neglect	10 (4.67%)
Lived in foster care	2 (0.93%)

*M*, Mean; *SD*, Standard deviation; *N*, Sample size; ACEs, Adverse Childhood Experiences. ^a^ Indicates that groups were not mutually exclusive.

**Table 2:** Descriptive statistics and Pearson’s *r* correlations among key study variables.

Variables	*M* (*SD*)	1	2	3	4	5	6
1. Mental Health Symptoms (BSI-GSI)	59.56 (12.92)	1					
2. Anxiety Symptoms (BSI)	55.73 (12.17)	0.86 ***	1				
3. Depression Symptoms (BSI)	59.27 (11.01)	0.88 ***	0.76 ***	1			
4. Adverse Childhood Experiences (ACEs)	2.20 (2.59)	0.37 ***	0.32 ***	0.31 ***	1		
5. Child–Dog Emotional Closeness (MDORS)	40.33 (7.59)	0.07	0.13	0.04	0.14	1	
6. Child–Pet Positive Interactions (CTAQ)	29.80 (4.27)	−0.03	0.03	−0.04	−0.01	0.60 ***	1
7. Benevolent Childhood Experiences (BCEs)	8.97 (1.57)	−0.38 ***	−0.30 ***	−0.30 ***	−0.62 ***	−0.06	0.03

*M*, Mean; *SD*, Standard Deviation; BSI, Brief Symptom Inventory; GSI, Global Severity Index; MDORS, Monash Dog-Owner Relationship Scale; CTAQ, Children’s Treatment Towards Animals Questionnaire; ***, *p* < 0.001.

**Table 3:** Model output of mental health symptoms (BSI-GSI) as a function of ACEs, BCEs, and child–pet relationships.

	Model 1—Child–Dog Emotional Closeness		Model 2—Child–Pet Positive Interactions		Model 3—Benevolent Childhood Experiences	
	*B (SE)*	*CI*	*B (SE)*	*CI*	*B (SE)*	*CI*
Demographics						
Constant	49.01 (8.11) ***	33.00–65.03	55.09 (7.40) ***	40.49–69.69	55.13 (6.86) ***	41.60–68.65
Age	0.43 (0.38)	−0.31–1.18	0.09 (0.34)	−0.58–0.76	0.16 (0.32)	−0.47–0.78
Female/Non-binary/Other (Male)	2.51 (2.52)	−2.47–7.49	3.16 (2.34)	−1.45–7.78	3.17 (2.12)	−1.02–7.35
Hispanic/Latinx (Non-Hispanic/Latinx/Prefer not to say)	1.24 (2.71)	−4.11–6.60	1.08 (2.49)	−3.84–6.00	1.17 (2.28)	−3.33–5.66
Main Effects and Interactions—Model 1 (*R*^2^ = 0.14)						
ACEs	1.81 (0.42) ***	0.99–2.64				
Child–Dog Emotional Closeness	0.01 (0.13)	−0.24–0.26				
ACEs x Child–Dog Emotional Closeness	−0.05 (0.06)	−0.17–0.06				
Main Effects and Interactions—Model 2 (*R*^2^ = 0.14)						
ACEs			1.75 (0.36) ***	1.05–2.46		
Child–Pet Positive Interactions			−0.12 (0.21)	−0.52–0.29		
ACEs x Child–Dog Positive Interactions			0.02 (0.08)	−0.14–0.18		
Main Effects and Interactions—Model 3 (*R*^2^ = 0.21)						
ACEs					1.35 (0.43) **	0.50–2.20
BCEs					−3.54 (0.77) ***	−5.06–−2.02
ACEs x BCEs					0.60 (0.17) ***	0.26–0.93

*B*, Unstandardized beta; *SE,* Standard error; *CI*, 95% confidence interval. Reference category is indicated in parentheses. Model 1 is based on a sample of *N* = 176 (those with pet dog in childhood), Model 2 is based on a sample of *N* = 203 (those with any pet in childhood), and Model 3 is based on a sample *N* = 214 (all participants). ** *p <* 0.01, *** *p* < 0.001.

## Data Availability

The data presented in this study are available on request.

## References

[1] Felitti V.J., Anda R.F., Nordenberg D., Williamson D.F., Spitz A.M., Edwards V., Marks J.S. (1998). Relationship of childhood abuse and household dysfunction to many of the leading causes of death in adults: The Adverse Childhood Experiences (ACE) Study. Am. J. Prev. Med..

[2] Merrick M.T., Ford D.C., Ports K.A., Guinn A.S. (2018). Prevalence of adverse childhood experiences from the 2011–2014 behavioral risk factor surveillance system in 23 states. JAMA Pediatrics.

[3] Anda R.F., Felitti V.J., Bremner J.D., Walker J.D., Whitfield C., Perry B.D., Dube S.R., Giles W.H. (2006). The enduring effects of abuse and related adverse experiences in childhood. Eur. Arch. Psychiatry Clin. Neurosci..

[4] Brown S.M., Rienks S., McCrae J.S., Watamura S.E. (2019). The co-occurrence of adverse childhood experiences among children investigated for child maltreatment: A latent class analysis. Child Abus. Negl..

[5] Cronholm P.F., Forke C.M., Wade R., Bair-Merritt M.H., Davis M., Harkins-Schwarz M., Pachter L.M., Fein J.A. (2015). Adverse childhood experiences: Expanding the concept of adversity. Am. J. Prev. Med..

[6] Petruccelli K., Davis J., Berman T. (2019). Adverse childhood experiences and associated health outcomes: A systematic review and meta-analysis. Child Abus. Negl..

[7] Moore K.A., Ramirez A.N. (2016). Adverse childhood experience and adolescent well-being: Do protective factors matter?. Child Indic. Res..

[8] McLaughlin K.A. (2016). Future directions in childhood adversity and youth psychopathology. J. Clin. Child Adolesc. Psychol..

[9] Narayan A.J., Rivera L.M., Bernstein R.E., Harris W.W., Lieberman A.F. (2018). Positive childhood experiences predict less psychopathology and stress in pregnant women with childhood adversity: A pilot study of the benevolent childhood experiences (BCEs) scale. Child Abus. Negl..

[10] Crandall A., Miller J.R., Cheung A., Novilla L.K., Glade R., Novilla M.L.B., Magnusson B.M., Leavitt B.L., Barnes M.D., Hanson C.L. (2019). ACEs and counter-ACEs: How positive and negative childhood experiences influence adult health. Child Abus. Negl..

[11] Bethell C., Jones J., Gombojav N., Linkenbach J., Sege R. (2019). Positive childhood experiences and adult mental and relational health in a statewide sample: Associations across adverse childhood experiences levels. JAMA Pediatrics.

[12] Crandall A., Broadbent E., Stanfill M., Magnusson B.M., Novilla M.L.B., Hanson C.L., Barnes M.D. (2020). The influence of adverse and advantageous childhood experiences during adolescence on young adult health. Child Abus. Negl..

[13] Gartland D., Riggs E., Muyeen S., Giallo R., Afifi T.O., MacMillan H., Herrman H., Bulford E., Brown S.J. (2019). What factors are associated with resilient outcomes in children exposed to social adversity? A systematic review. BMJ Open.

[14] Melkman E.P. (2017). Childhood adversity, social support networks and well-being among youth aging out of care: An exploratory study of mediation. Child Abus. Negl..

[15] Zilcha-Mano S., Mikulincer M., Shaver P.R. (2012). Pets as safe havens and secure bases: The moderating role of pet attachment orientations. J. Res. Personal..

[16] Melson G.F., Schwarz R.L., Beck A.M. (1997). Importance of companion animals in children’s lives: Implications for veterinary practice. J. Am. Vet. Med Assoc..

[17] Beetz A., Julius H., Turner D., Kotrschal K. (2012). Effects of social support by a dog on stress modulation in male children with insecure attachment. Front. Psychol..

[18] Purewal R., Christley R., Kordas K., Joinson C., Meints K., Gee N., Westgarth C. (2017). Companion animals and child/adolescent development: A systematic review of the evidence. Int. J. Environ. Res. Public Health.

[19] Bodsworth W., Coleman G. (2001). Child–companion animal attachment bonds in single and two-parent families. Anthrozoös.

[20] Westgarth C., Boddy L.M., Stratton G., German A.J., Gaskell R.M., Coyne K.P., Bundred P., McCune S., Dawson S. (2013). Pet ownership, dog types and attachment to pets in 9–10 year old children in Liverpool, UK. BMC Vet. Res..

[21] Carr S., Rockett B. (2017). Fostering secure attachment: Experiences of animal companions in the foster home. Attach. Hum. Dev..

[22] Strand E.B. (2004). Interparental conflict and youth maladjustment: The buffering effects of pets. Stress Trauma Crisis.

[23] Applebaum J.W., Zsembik B.A. (2020). Pet Attachment in the Context of Family Conflict. Anthrozoös.

[24] Barlow M.R., Hutchinson C.A., Newton K., Grover T., Ward L. (2012). Childhood neglect, attachment to companion animals, and stuffed animals as attachment objects in women and men. Anthrozoös.

[25] Hawkins R.D., McDonald S.E., O’Connor K., Matijczak A., Ascione F.R., Williams J.H. (2019). Exposure to intimate partner violence and internalizing symptoms: The moderating effects of positive relationships with pets and animal cruelty exposure. Child Abus. Negl..

[26] McDonald S.E., O’Connor K., Matijczak A., Murphy J., Applebaum J.W., Tomlinson C.A., Wike T.L., Kattari S.K. Victimization and psychological wellbeing among sexual and gender minority emerging adults: Testing the moderating role of emotional comfort from companion animals. J. Soc. Soc. Work Res..

[27] Kerns K.A., Stuart-Parrigon K.L., Coifman K.G., van Dulmen M.H., Koehn A. (2018). Pet Dogs: Does their presence influence preadolescents’ emotional responses to a social stressor?. Soc. Dev..

[28] Kertes D.A., Liu J., Hall N.J., Hadad N.A., Wynne C.D., Bhatt S.S. (2017). Effect of pet dogs on children’s perceived stress and cortisol stress response. Soc. Dev..

[29] Karatekin C., Ahluwalia R. (2020). Effects of adverse childhood experiences, stress, and social support on the health of college students. J. Interpers. Violence.

[30] Blum R.W., Li M., Naranjo-Rivera G. (2019). Measuring adverse child experiences among young adolescents globally: Relationships with depressive symptoms and violence perpetration. J. Adolesc. Health.

[31] Lee H., Kim Y., Terry J. (2020). Adverse childhood experiences (ACEs) on mental disorders in young adulthood: Latent classes and community violence exposure. Prev. Med..

[32] Boulet J., Boss M.W. (1991). Reliability and validity of the Brief Symptom Inventory. Psychol. Assess. J. Consult. Clin. Psychol..

[33] Drobnjak S., Gellman M.D., Turner J.R. (2013). Brief Symptom Inventory. Encyclopedia of Behavioral Medicine.

[34] McDonald S.E., Vidacovich C., Ascione F.R., Williams J.H., Green K.E. (2015). The children’s treatment of animals questionnaire: A rasch analysis. Anthrozoos.

[35] Dwyer F., Bennett P.C., Coleman G.J. (2006). Development of the Monash dog owner relationship scale (MDORS). Anthrozoös.

[36] Faul F., Erdfelder E., Buchner A., Lang A.-G. (2009). Statistical power analyses using G* Power 3.1: Tests for correlation and regression analyses. Behav. Res. Methods.

[37] Makriyianis H.M., Adams E.A., Lozano L.L., Mooney T.A., Morton C., Liss M. (2019). Psycholoical inflexibility mediates the relationship between adverse childhood experiences and mental health outcomes. J. Contextual Behav. Sci..

[38] Cohrdes C., Mauz E. (2020). Self-Efficacy and emotional stability buffer negative effects of adverse childhood experiences on young adult health-related quality of life. J. Adolesc. Health.

[39] Sheridan M.A., McLaughlin K.A. (2014). Dimensions of early experience and neural development: Deprivation and threat. Trends Cogn. Sci..

[40] Schilling E.A., Aseltine R.H., Gore S. (2008). The impact of cumulative childhood adversity on young adult mental health: Measures, models, and interpretations. Soc. Sci. Med.

[41] McLaughlin K.A., Sheridan M.A. (2016). Beyond cumulative risk: A dimensional approach to childhood adversity. Curr. Dir Psychol. Sci..

[42] McPhedran S. (2009). Animal abuse, family violence, and child wellbeing: A review. J. Fam. Violence.

[43] Haden S., McDonald S.E., Murphy J., Shackelford T. (2020). Violence against family pets. SAGE Handbook of Domestic Violence.

[44] Murphy J.L., Voorhees E.V., O’Connor K.E., Tomlinson C.A., Matijczak A., Applebaum J.W., Ascione F.R., Williams J.H., McDonald S.E. Positive engagement with pets buffers the impact of intimate partner violence on callous-unemotional traits in children. J. Interpers. Violence.

[45] Pendry P., Vandagriff J.L. (2020). Salivary Studies of the Social Neuroscience of Human–Animal Interaction. Salivary Bioscience.

[46] McDonald S.E., Tomlinson C.A., Applebaum J.W., Moyer S.W., Brown S.M., Carter S., Kinser P.A. (2021). Human–Animal Interaction and Perinatal Mental Health: A Narrative Review of Selected Literature and Call for Research. Int. J. Environ. Res. Public Health.

[47] Carter C.S., Porges S.W., Freund L.S., McCune S., Esposito L., Gee N.R., McCardle P. (2016). Neural mechanisms underlying human-animal interaction: An evolutionary perspective. The Social Neuroscience of Human-Animal Interaction.

[48] Tomlinson C.A., Murphy J.L., Williams J.M., Hawkins R.D., Matijczak A., Applebaum J.W., McDonald S.E. (2021). Testing the moderating role of victimization and microaggressions on the relationship between human-animal interaction and psychological adjustment among LGBTQ+ emerging adults. Hum.-Anim. Interact. Bull..

[49] Susser E., Widom C.S. (2012). Still searching for lost truths about the bitter sorrows of childhood. Schizophr. Bull..

